# Tree-based ensemble machine learning models in the prediction of acute respiratory distress syndrome following cardiac surgery: a multicenter cohort study

**DOI:** 10.1186/s12967-024-05395-1

**Published:** 2024-08-15

**Authors:** Hang Zhang, Dewei Qian, Xiaomiao Zhang, Peize Meng, Weiran Huang, Tongtong Gu, Yongliang Fan, Yi Zhang, Yuchen Wang, Min Yu, Zhongxiang Yuan, Xin Chen, Qingnan Zhao, Zheng Ruan

**Affiliations:** 1 Department of Thoracic Surgery, Shanghai General Hospital, Shanghai Jiao Tong University School of Medicine, No. 650 Xinsongjiang Road, Shanghai, 201620 China; 2 Department of Cardiovascular Surgery, Shanghai General Hospital, Shanghai Jiao Tong University School of Medicine, No. 85 Wujin Road, Shanghai, 200080 China; 3 Qing Yuan Research Institute, SEIEE, Shanghai Jiao Tong University, No. 800 Dongchuan Road, Shanghai, 200240 China; 4 Department of Pharmacy, Shanghai Sixth People’s Hospital Affiliated to Shanghai Jiao Tong University School of Medicine, No. 600 Yishan Road, Shanghai, 200233 China; 5 Department of Thoracic and Cardiovascular Surgery, Nanjing First Hospital, Nanjing Medical University, No. 68 Changle Road, Nanjing, 210006 China; 6 Department of Clinical Pharmacy, Shanghai General Hospital, Shanghai Jiao Tong University School of Medicine, No. 650 Xinsongjiang Road, Shanghai, 201620 China

**Keywords:** Acute respiratory distress syndrome, Cardiac surgery, Machine learning, Prediction model, SHAP value

## Abstract

**Background:**

Acute respiratory distress syndrome (ARDS) after cardiac surgery is a severe respiratory complication with high mortality and morbidity. Traditional clinical approaches may lead to under recognition of this heterogeneous syndrome, potentially resulting in diagnosis delay. This study aims to develop and external validate seven machine learning (ML) models, trained on electronic health records data, for predicting ARDS after cardiac surgery.

**Methods:**

This multicenter, observational cohort study included patients who underwent cardiac surgery in the training and testing cohorts (data from Nanjing First Hospital), as well as those patients who had cardiac surgery in a validation cohort (data from Shanghai General Hospital). The number of important features was determined using the sliding windows sequential forward feature selection method (SWSFS). We developed a set of tree-based ML models, including Decision Tree, GBDT, AdaBoost, XGBoost, LightGBM, Random Forest, and Deep Forest. Model performance was evaluated using the area under the receiver operating characteristic curve (AUC) and Brier score. The SHapley Additive exPlanation (SHAP) techinque was employed to interpret the ML model. Furthermore, a comparison was made between the ML models and traditional scoring systems. ARDS is defined according to the Berlin definition.

**Results:**

A total of 1996 patients who had cardiac surgery were included in the study. The top five important features identified by the SWSFS were chronic obstructive pulmonary disease, preoperative albumin, central venous pressure_T4, cardiopulmonary bypass time, and left ventricular ejection fraction. Among the seven ML models, Deep Forest demonstrated the best performance, with an AUC of 0.882 and a Brier score of 0.809 in the validation cohort. Notably, the SHAP values effectively illustrated the contribution of the 13 features attributed to the model output and the individual feature's effect on model prediction. In addition, the ensemble ML models demonstrated better performance than the other six traditional scoring systems.

**Conclusions:**

Our study identified 13 important features and provided multiple ML models to enhance the risk stratification for ARDS after cardiac surgery. Using these predictors and ML models might provide a basis for early diagnostic and preventive strategies in the perioperative management of ARDS patients.

**Supplementary Information:**

The online version contains supplementary material available at 10.1186/s12967-024-05395-1.

## Background

Cardiac surgery is increasingly frequent in a growing population of elderly patients with multiple morbidities in both developed and developing countries. A severe complication of cardiac surgery is acute respiratory distress syndrome (ARDS). ARDS is the leading cause of postoperative hypoxic respiratory failure, substantially increasing perioperative and long-term mortality [1, 2]. Data from an international, observational, multicenter prospective cohort investigation, namely the LUNG SAFE study, revealed that the severe forms of ARDS had a death rate of up to 46% [3]. Survivors of ARDS are also likely to suffer from long-term physical and psychological morbidity [4].

A previous systematic review reported that the incidence of ARDS after cardiac surgery varied from 0.4 to 8.1% [5]. This variability could be attributed to differences in study populations and definitions used for ARDS. In 2012, the Berlin definition was published, and it described ARDS as having an acute onset with diffuse bilateral pulmonary infiltrates that cannot be explained by cardiac failure and/or fluid overload [6]. However, impacted by cardiovascular comorbidities, it is likely that ARDS after cardiac surgery has a poorer prognosis compared with ARDS from other causes. As a consequence, the occurrence of ARDS after cardiac surgery is more common and relatively challenging to diagnose. When patients present with evident hypoxemic and positive radiographic findings, it often indicates that ARDS has progressed for some time [7]. In other words, the Berlin definition cannot detect ARDS in the early stages, potentially leading to delays in diagnosis and treatment. The failure of clinicians to recognize ARDS is a barrier to the use of early prevention strategies, including lung-protective ventilation, conservative fluid management, and adjunctive measures proven to improve survival, such as prone positioning [8–10]. However, timely diagnosis of ARDS is not an easy task. On the one hand, clinicians have limited ability to distinguish ARDS from other causes of respiratory failure. It is often challenging to diagnose ARDS in patients with similar symptoms of potential clinical conditions, particularly in patients who have undergone cardiopulmonary bypass (CPB). The interpretation of a chest radiograph may be influenced by a number of comorbidities, such as pulmonary congestion, lung infection, chest tubes and or pleural effusions, and cardiac dysfunction [7, 11]. On the other hand, owing to the multifactorial and intricate pathogenesis of ARDS, few biomarkers have been proven to be effective in assessing risk [12]. Therefore, developing a clinical decision tool to assist clinicians accurately and early predict ARDS is a valuable and crucial.

For over a decade, prediction models have been proposed for use in clinical practice. Several studies have used clinical scoring systems to evaluate the risk of ARDS in diverse clinical settings, such as ARDS, Lung Injury Prediction Study (LIPS), and Surgical Lung Injury Prediction (SLIP) scores [13–15]. However, these models have certain inherent limitations. Methodologically, the scoring systems are constructed using the traditional logistic regression method, which requires statistical assumptions regarding a linear relationship between covariates and outcomes that are known to be clinically relevant. In addition, most studies derived models on the basis of small-scale candidate covariate pools, using the traditional approach, that involves initial univariate analysis to select variables followed by multivariate regression, potentially leading to underfitting and low classification accuracy. Furthermore, these models were not specifically designed for perioperative cardiac patients in terms of etiology, making it difficult to use them to evaluate ARDS in high-risk cardiac surgery.

With the advancements in electronic health records (EHR) and diverse datasets, machine learning (ML) algorithms are increasingly employed to help diagnose and prevent specific diseases. As a new analysis tool, ML has the advantages of higher prediction accuracy, handling larger datasets, and analyzing more complex non-linear association between covariates and clinical outcomes. Several ML techniques have been applied to assess ARDS risk. For example, a novel gradient-boosting tree model could predict ARDS with high-precision prediction (within 48 h before its onset) [16]. Cardiac surgery with CPB induces unique physiological perturbations, resulting in different pathological and physiological abnormalities associated with ARDS compared to other causes. To our knowledge, there is limited research using ML to assess ARDS risk after CPB cardiac surgery. In this study, we aimed to (i) develop and validate seven tree-based ML models, by incorporating diverse perioperative data, to predict ARDS in patients who had cardiac surgery; (ii) investigate the performance of six conventional scoring systems to determine whether they can be used to predict ARDS after cardiac surgery.

## Method

### Participants

This is a multicenter, retrospective, observational cohort study. We enrolled in-patients who had coronary artery bypass grafting (CABG), valvular surgery, or a combination of both treatments from two academic medical centers in China: Shanghai General Hospital, Shanghai Jiao Tong University School of Medicine, Shanghai (SHGH) and Nanjing First Hospital Affiliated to Nanjing Medical University, Nanjing (NFH). Patients were excluded if they met any of the following criteria: (i) younger than 18 years; (ii) preoperative ARDS; (iii) did not receive CPB; (iv) mechanical ventilation before surgery; (v) trauma, sepsis, aspiration, shock, and respiratory failure at any point during hospitalization before surgery.

### Data source and partitioning

We developed prediction models based on the patient cohort from NFH, which comprised 1493 patients who had cardiac surgery between January 2016 and December 2021. They were randomly assigned to training and testing cohorts in a 7:3 ratio. To further assess the performance of the models, data from SHGH were used for subsequent external validation. We curated data between January 2016 and December 2022, using the same recruitment criteria and included 503 patients in the validation dataset. As the proportion of positive cases in the consecutive cardiac patient cohort was relatively small, in order to increase ARDS cases and reduce modeling bias, we created ARDS cohorts which recruited patients admitted to the two centers between January 2013 and December 2022. Five physician investigators (NFH cohort: Hang Zhang, Hong Lang, Wuwei Wang, and Yunzhang Wu; SHGH cohort: Dewei Qian) gathered and organized the databases. Data integrity was verified by a secondary manual review of the EHR.

This study was approved by the Medical Ethical Committee of SHGH and NFH. Written informed consent from participants was waived because of the retrospective nature of the study. This study was conducted in accordance with the Helsinki Declaration (1964) and was reported according to the Transparent Reporting of a Multivariable Prediction Model for Individual Prognosis or Diagnosis (TRIPOD) guideline [17].

### Clinical information and airway management

In the model development phase, we selected a large set of clinical characteristics including baseline information (patient demographics, comorbidities, and admission assessment), medical text, imaging data, laboratory biomarkers, medication, treatment detail, and CPB data. In the validation phase, only the important features were collected and incorporated into the models.

All patients were performed with the same standard surgical procedure and similar anesthesia induction and maintenance. The standard intensive care unit (ICU) protocols were approximately similar in two centers. In brief, postoperatively, patients were placed on mechanical ventilation in synchronized intermittent mandatory ventilation or assist/control models, set at 8–10 mL/kg tidal volume and 5 cmH_2_O positive end-expiratory pressure (PEEP). Arterial blood gas (ABG) analysis was checked at regular intervals ranging from every 30 min to 6 h, depending on the patient’s condition. Patients were extubated after meeting the following criteria: (i) alertness and cooperation, with sufficient muscle strength; (ii) hemodynamic stability, without low cardiac output syndrome or signs of myocardial ischemia, requiring minimal muscular support (norepinephrine or epinephrine ≤ 0.05 μg/kg/min); (iii) chest tube drainage < 50 mL/h, without active bleeding; (iv) acceptable ABG, in the absence of respiratory distress, with FiO_2_ ≤ 0.5 and PEEP ≤ 5 cmH_2_O, including PaO_2_ ≥ 80 mmHg and PaCO_2_ < 45 mmHg.

### End point definition

ARDS is defined according to the 2012 Berlin definition, specifically, the partial pressure of arterial oxygen and fraction of inspired oxygen (PaO_2_/FiO_2_) less than 300 mmHg, diffuse bilateral pulmonary infiltration, and pulmonary artery wedge pressure less than 18 mmHg, in absence of hydrostatic or cardiogenic pulmonary edema [6].

### Model development

This study mainly comprised three stages: (i) feature selection, (ii) model derivation and validation, and (iii) model comparison. First, we applied sliding windows sequential forward feature selection (SWSFS), a ML-based feature selection method, to determine the number of important features. Based on the selected features, we constructed a set of tree-based ML models. Finally, in the testing and validation cohorts, we compared the model performance with other six conventional scoring systems: The Logistic Organ Dysfunction System (LODS), Sequential Organ Failure Assessment (SOFA), LIPS, SLIP, Multiple Organ Dysfunction Score (MODS), and ARDS scores [13–15, 18–20].

### Feature selection

We selected features using the SWSFS method. Details of the SWSFS have been described previously [21, 22]. Briefly, the feature importance score (FIS) was evaluated by the Gini index which was initially obtained using a Random Forest (RF) classifier via the *importance* function in R. To minimize the random error, we ran the RF 30 times and calculated the average Gini index of each feature. FIS was ranked in descending order and included one by one to the RF model. Afterward, we plotted the 'out of bagging (OOB) ', a parameter of evaluating the model error, which measured the performance of each model consisting of a certain number of features. Based on the lowest OOB, the number of important features was determined. Finally, these features were used as input variables to construct the ML models.

### ML models

We developed the prediction models using the following model-building approaches, which are commonly used and advanced tree-based learning algorithms for binary classification: Decision Tree, Gradient Boosting Decision Tree (GBDT), eXtreme Gradient Boosting (XGBoost), Adaptive Boosting (AdaBoost), Light Gradient Boosting Machine (LightGBM), RF, and Deep Forest (DF). Except for Decision Tree, all of the algorithms are tree-based ensemble learning algorithms. The ensemble learning methods have the advantage of combining predictions from multiple ML models to achieve more accurate and robust predictive performance. In addition, they are particularly useful in reducing overfitting, where a model performs well in training data but fails to replicate the results in testing data. The detailed introduction of ML algorithms is provided in the Additional file 1.

### Model interpretation

Understanding why a model made a prediction is important for clinical application. We used SHapley Additive exPlanation (SHAP) values to demonstrate the impact of features on ARDS risk prediction. SHAP, inspired by the cooperative game theory, develops an additive explanatory model that considers all features as contributors [23]. The model calculates a predictive value for each predictive sample, assigning the SHAP to each feature. This technique could examine the significance of each feature on individual or global model output, making it easy to understand the contribution of individual features to outcome. As an example, we used SHAP values, by employing the SHAP plot function, to reveal the complex relationship between features and output in the RF model.

### Statistical analysis

For descriptive analysis, continuous variables are reported as means (SD) and categorical variables as frequencies and proportions. Differences between ARDS or non-ARDS cohort were tested using *t* test, Mann–Whitney *U* test, chi-square test, or Fisher's exact probability method as appropriate. As all variables are collected by manual review of the EHR from two centers, missing values are unavoidable. Out datasets had missing data ranging from 0 to 3.15%. We used the multiple imputation method, by employing the “mice” package in R, to impute missing data. For the imbalance of data (the imbalance between positive and negative cases) in the training cohort, we used the random over-sampling method to prevent overfitting. Model performance was evaluated according to a range of ML metrics including the area under the receiver operating characteristic curve (AUC), Brier score, sensitivity, specificity, positive predictive value, negative predictive value, and F1 score. Statistical analyses were performed using R (version 4.0.3) and Python (version 3.8). A two-sided P value < 0.05 was considered statistically significant.

## Results

### Characteristics of the study population

The study populations in the training, testing, and validation cohorts numbered 1057, 436, and 503, respectively. In the discovery phase, the study population comprised 1493 patients from NFH. The mean (SD) age of the NFH cohort was 61.9 (10.5) years, with 867 (58.1%) being males. In both training and testing cohorts, a comparison of patients with or without ARDS is presented in Additional file 2: Table S1. The dataset for the validation phase comprised 503 patients from SHGH. The mean age of these patients was 63.1 (11.6) years, with 308 (61.2%) being males. The occurrences of ARDS in the training, testing, and validation cohorts were 11.5%, 11.9%, and 15.1%, respectively. The flow chart for the patient selection process and data partitioning is provided in Additional file 2: Fig. S1.

### Feature importance

**Fig. 1:**
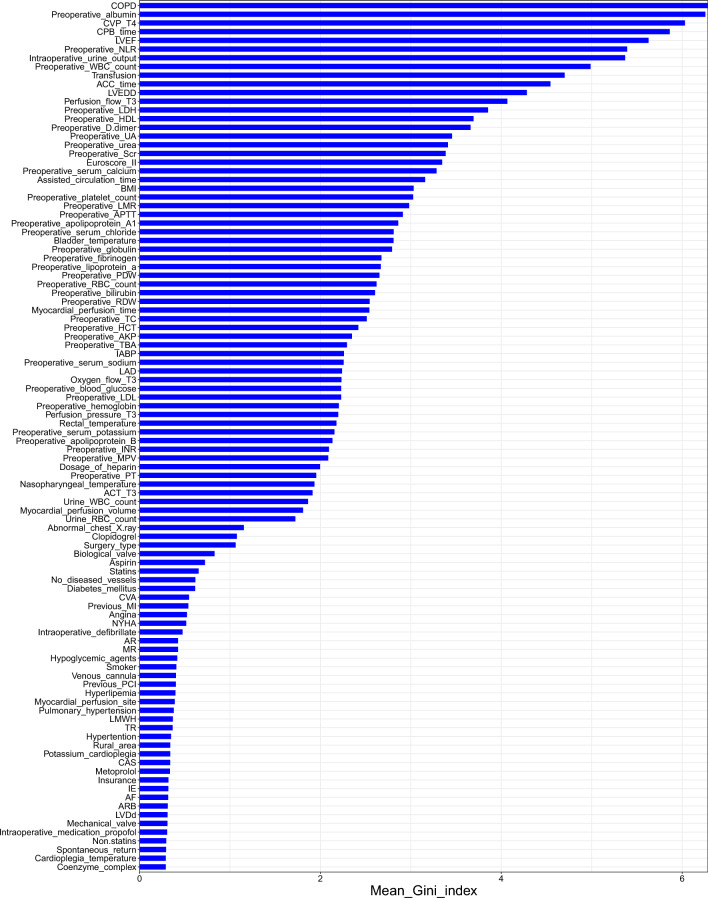
Importance matrix plot of candidate features in the training cohort

**Fig. 2:**
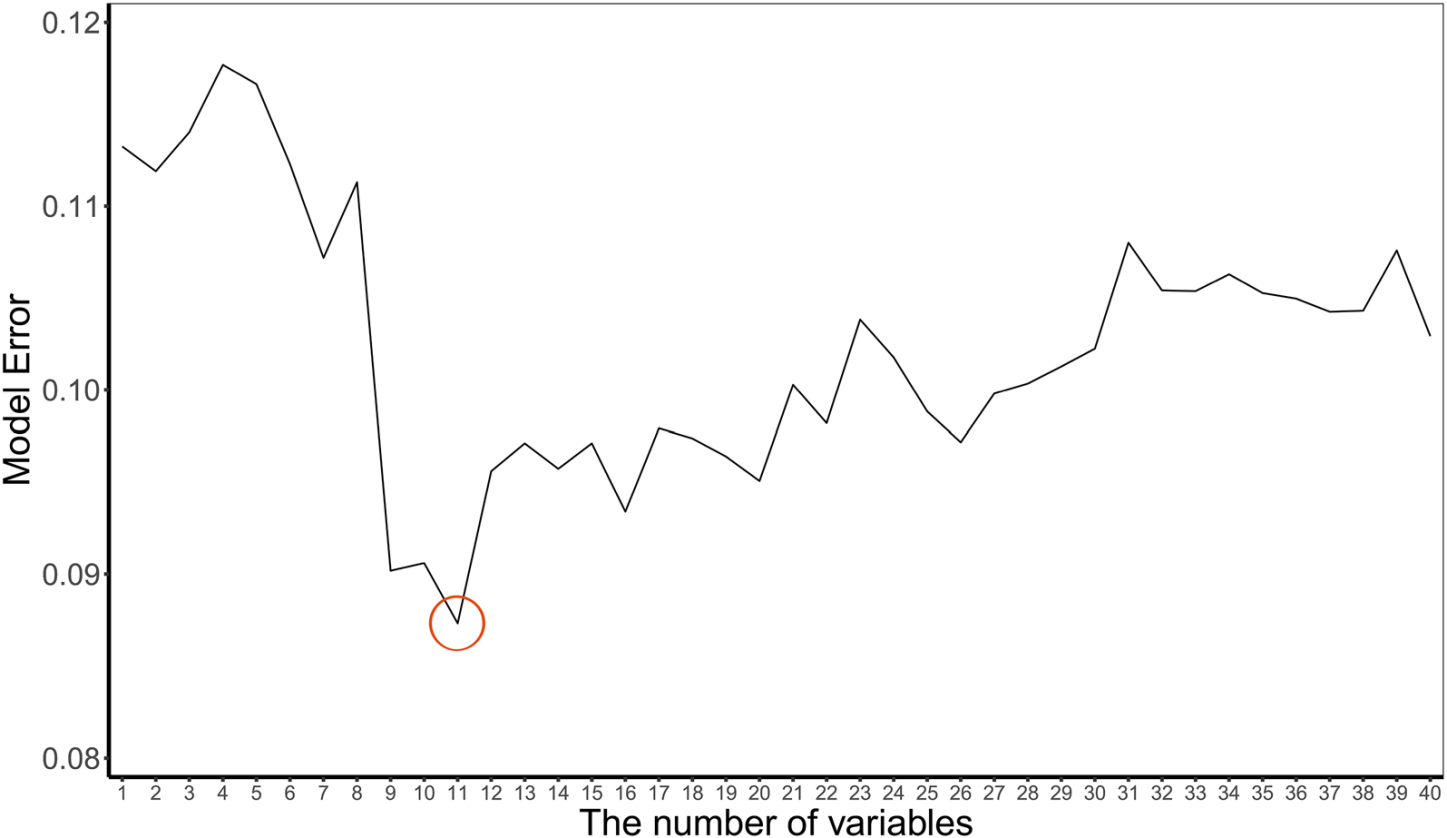
Feature selection using the sliding windows sequential forward feature selection method (SWSFS). The SWSFS was used to determine a set of important features of ARDS. First, the candidate features were included one by one in the Random Forest model in order of their rank in the importance matrix. Then, the optimal number of features (13 features) was determined by minimum out of bag error (red circle)

In the training cohort, 126 perioperative features were included in the model and ranked in descending order (Fig. 1). The importance matrix plot showed the top 100 features of ARDS. The SWSFS identified 13 important features associated with the development of ARDS, including six preoperative features [chronic obstructive pulmonary disease (COPD), preoperative albumin, left ventricular ejection fraction (LVEF), left ventricular end-diastolic diameter (LVEDD), preoperative white blood cell (WBC) count, preoperative neutrophil-to-lymphocyte ratio (NLR)] and five intraoperative features [CPB time, aortic cross-clamping (ACC) time, transfusion, central venous pressure (CVP)_T4, intraoperative urine output] (Fig. 2). A comparison of important features of patients in the training, testing, and validation cohorts is provided in Additional file 2: Table S2.

### ML model and model performance

**Fig. 3:**
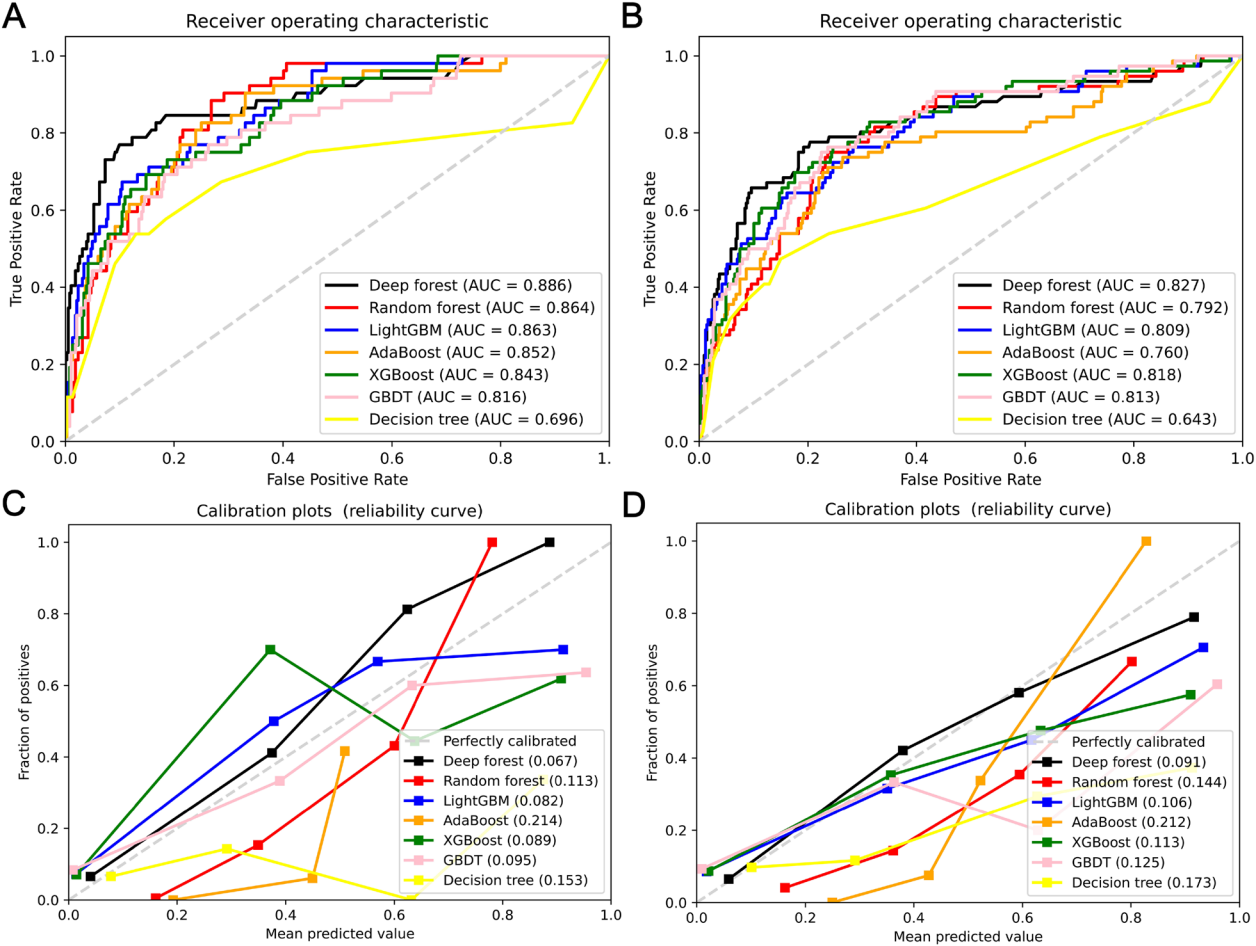
Performance of the machine models for predicting ARDS after cardiac surgery. Comparison of area under the receiver operating characteristic curves among Decision Tree, GBDT, XGBoost, AdaBoost, LightGBM, Random Forest, and Deep Forest in the testing and validation cohorts (**A** and **B**). Calibration plots of the models in the testing and validation cohorts (**C** and **D**)

In the testing and validation cohorts, we presented the AUC and Brier score for ML models. The DF model exhibited the highest AUC (0.886 [95% CI 0.831–0.940] in the testing cohort and 0.827 [95% CI 0.769–0.885] in the validation cohort), indicating the highest discrimination ability for ARDS classification; followed by RF model (0.864 [95% CI 0.820–0.909] and 0.792 [95% CI 0.736–0.848]), LightGBM (0.863 [95% CI 0.812–0.913] and 0.809 [95% CI 0.752–0.865]), AdaBoost (0.852 [95% CI 0.798–0.906] and 0.760 [95% CI 0.696–0.824]), XGBoost (0.843 [95% CI 0.788–0.897] and 0.818 [95% CI 0.764–0.873]), GBDT (0.816 [95% CI 0.753–0.880] and 0.813 [95% CI 0.759–0.867]), and Decision Tree (0.696 [95% CI 0.598–0.794] and 0.643 [95% CI 0.562–0.724]) (Fig. 3A, B). Accordingly, the DF model demonstrated the best calibration performance, with Brier scores of 0.067 and 0.091 in the testing and validation cohorts, respectively (Fig. 3C, D). Furthermore, a set of interpretable parameters (sensitivity, specificity, positive predictive value, negative predictive value, and F1 score) were calculated to evaluate the performance of the ML models (Table 1).

**Table 1:** Evaluation parameters of machine learning models in the testing and validation cohorts

ML models	Specificity (%)	Sensitivity (%)	PPV (%)	NPV (%)	F1 score
Testing cohort					
Decision Tree	84.6	53.8	32.1	93.1	0.80
GBDT	97.3	32.6	62.9	91.4	0.89
XGBoost	96.6	32.6	56.6	91.3	0.88
AdaBoost	89.0	57.6	41.6	93.9	0.85
LightGBM	97.9	34.6	69.2	91.7	0.90
Random Forest	92.4	46.1	45.2	92.6	0.86
Deep Forest	99.2	36.5	86.3	92.0	0.91
Validation cohort					
Decision Tree	85.0	47.3	36.0	90.0	0.79
GBDT	93.6	40.7	53.4	89.8	0.85
XGBoost	93.4	43.4	54.0	90.2	0.85
AdaBoost	77.9	64.4	34.2	92.5	0.75
LightGBM	95.0	43.4	61.1	90.4	0.87
Random Forest	87.3	44.7	38.6	89.8	0.80
Deep Forest	96.0	43.4	66.0	90.5	0.88

*ML* machine learning, *PPV* positive predictive value, *NPV* negative predictive value, *GBDT* Gradient Boosting Decision Tree, *AdaBoost* Adaptive Boosting, *LightGBM* Light Gradient Boosting Machine

**Fig. 4:**
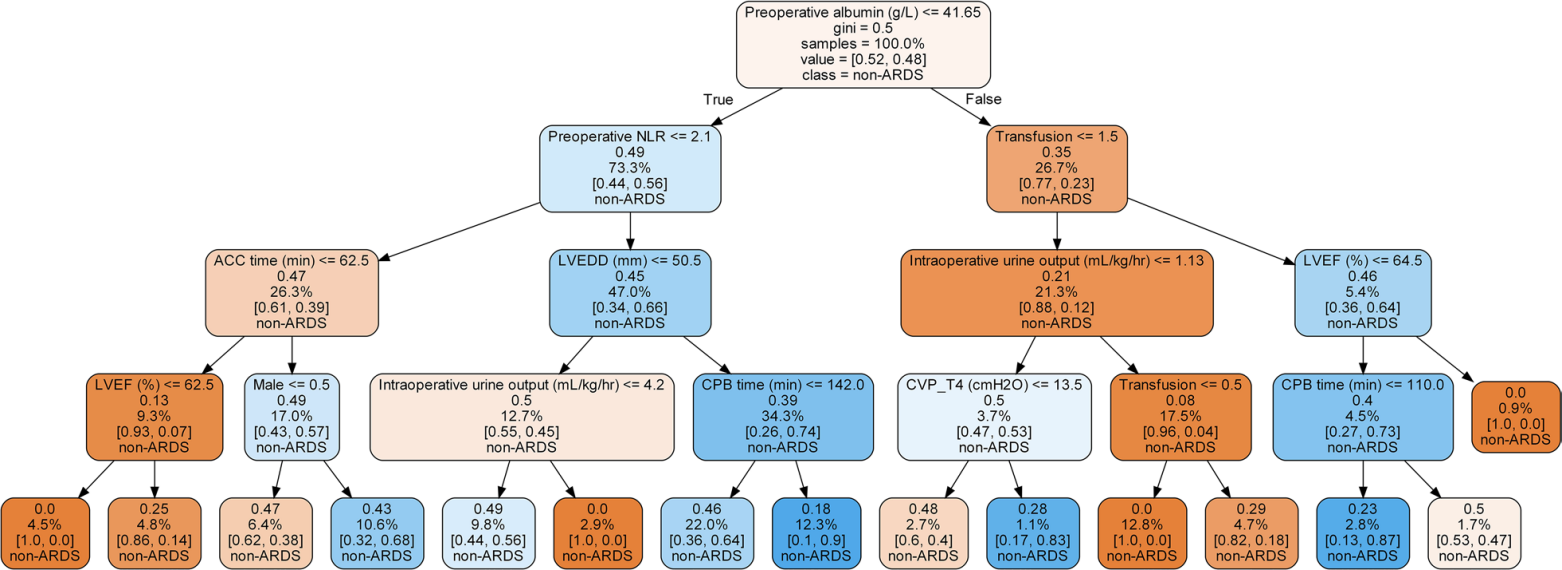
The decision tree results from the learning process for a specific patient without ARDS. Each node contains the proportion of samples considered up to that point in the tree and their distribution among the two different classes. Each leaf node is labeled with the classification choice. The darker the color of a leaf node, the higher the risk of being allocated to the non-ARDS class

In this study, the Decision Tree algorithm provided the fundamental framework for the ensemble learning models. We additionally presented a Decision Tree model for classifying patients into having ARDS or not. The higher the Gini index in the terminal leaf, the poorer the prediction accuracy. Five of the 14 terminal leaf nodes exceeded 0.40, indicating that the classification was inaccurate (Fig. 4).

### SHAP value visualization

**Fig. 5:**
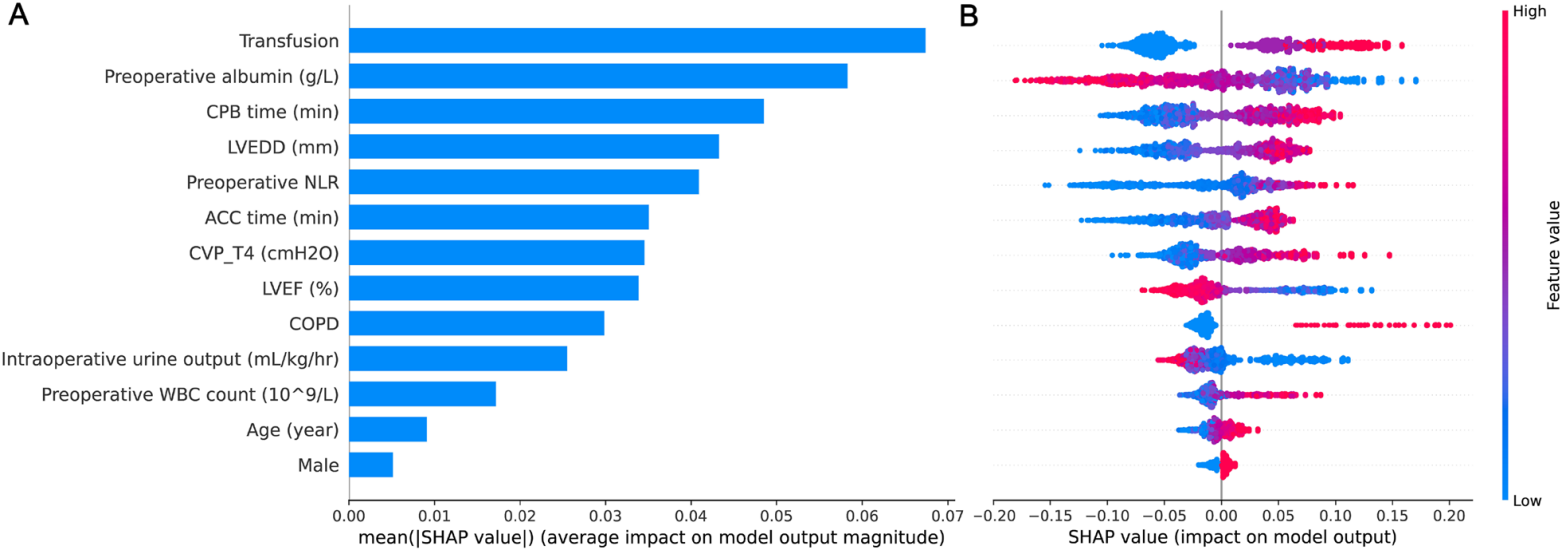
Importance evaluation in the SHAP summary analysis. **A** The plots show the 13 important features ranked in descending order in the SHAP analysis; **B** dot estimation of each feature on the model output. Each point represents an individual patient’s prediction on the model output. Red indicates higher SHAP values for specific features, while blue indicates lower SHAP values for specific features. The higher the SHAP value, the higher the risk of developing ARDS

We used the SHAP value to accurately represent the contribution of each feature to ARDS in the RF model. As depicted in the SHAP summary plot, the important features were ranked in descending order according to their contribution to model output. The top five features in the SHAP model were transfusion, preoperative albumin, CPB time, LVEDD, and preoperative NLR. Accordingly, using point estimation, we can clearly observe whether a feature has a negative impact on the prediction of non-ARDS or a positive impact on the prediction of ARDS (Fig. 5).

**Fig. 6:**
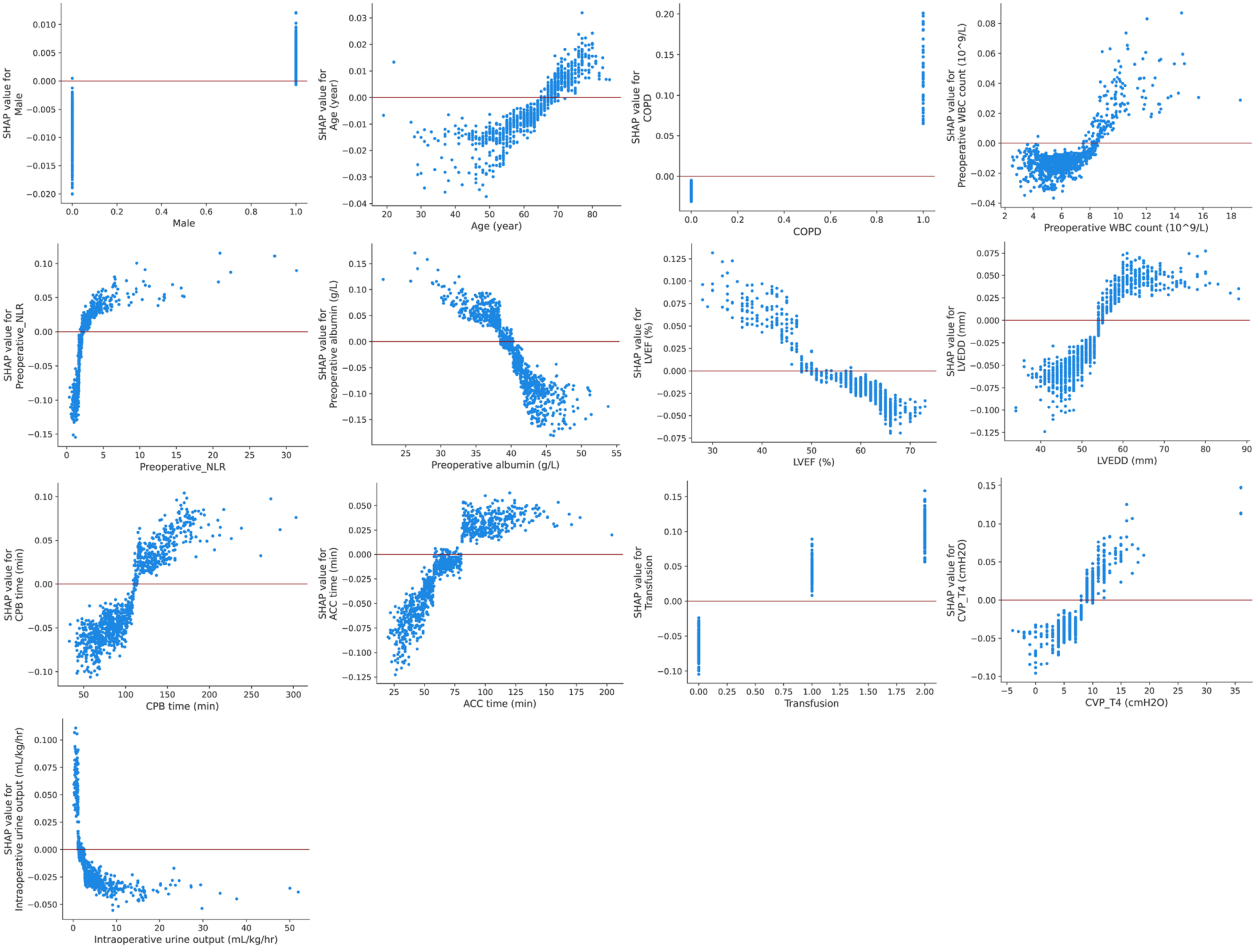
SHAP dependence plot of the Random Forest model. Each panel demonstrates that each feature affects the output of the Random Forest prediction model. The x-axis represents the raw values of each feature and the y-axis indicates the SHAP values of features. When the SHAP value of a specific feature exceeds zero, it indicates an increased risk of ARDS

**Fig. 7:**
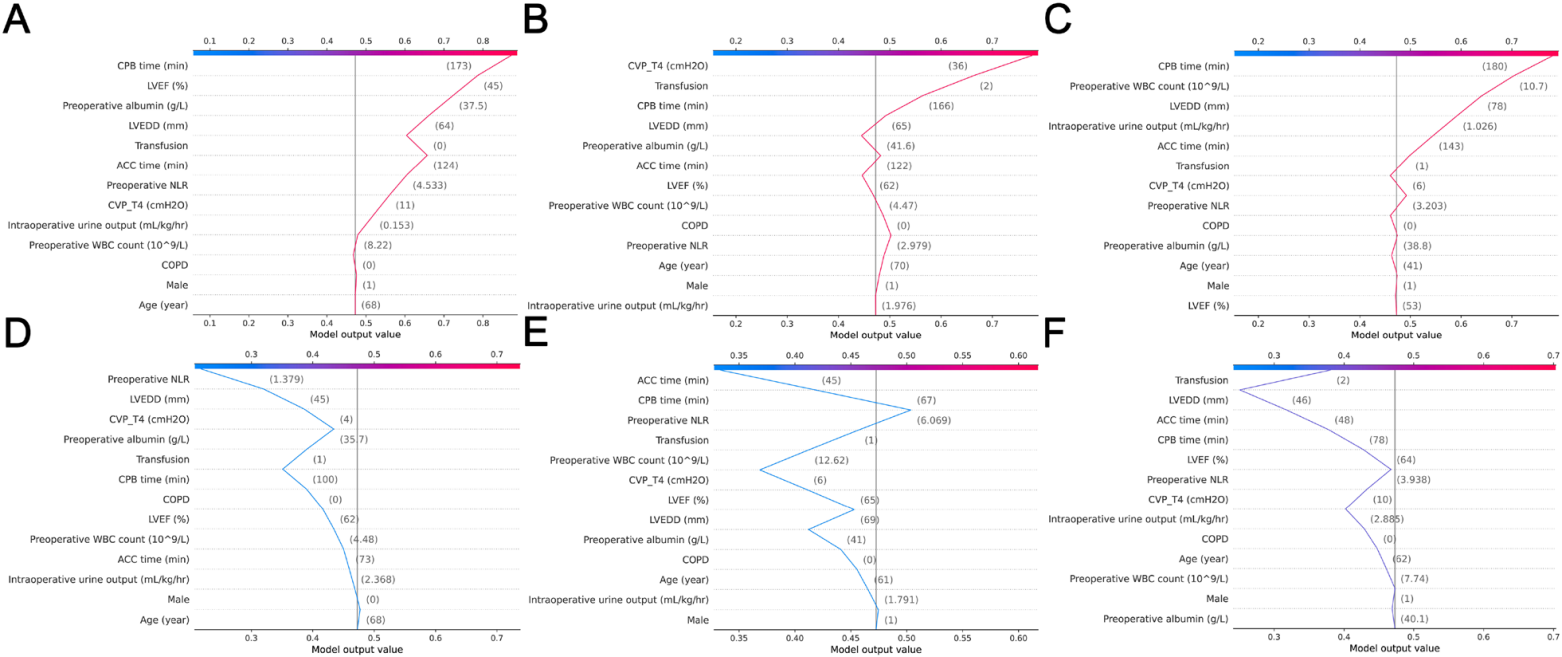
Individual patient decision path in the SHAP decision analysis. These plots depict the individual patient decision paths for predicting ARDS. All patients start with an average predicted value and are evaluated at each feature level to obtain the predicted probability of ARDS. **A–C** illustrate three patients predicted as ARDS while **D–F** show examples of three patients predicted as non-ARDS

The SHAP dependent plot was utilized to visualize the individual impact of features on ARDS risk predictions. When the SHAP value of a specific feature exceeds the zero threshold, it indicates an increased risk of ARDS. Conversely, falling below the zero threshold indicates a decreased risk of ARDS (Fig. 6). Additionally, we used the SHAP decision plot to display the individual patient-level predictions (Additional file 2: Fig. S2). This function provides a valuable depiction to describe the decision pathway for individual patient prediction and provides insights into why some patients (A–C) were predicted to have ARDS while others (D–F) were not (Fig. 7).

### Model comparison

The derivation detail for the ML models and the other six models is provided in Additional file 2: Table S3. In the testing and validation cohorts, the clinical scoring systems exhibited insufficient discrimination for predicting ARDS, with AUCs ranging from 0.613 to 0.708; nevertheless, their calibration performance is comparable to that of the ML models (Brier score 0.099–0.128) (Additional file 2: Table S4 and Fig. S3).

## Discussion

In this study, using clinical features retrieved from EHR, we developed and validated seven ML models for predicting ARDS after cardiac surgery. The ML models performed well in an external validation cohort, with AUC values ranging from 0.652 to 0.827. Our study demonstrated that the tree-based ensemble models outperformed conventional linear regression modeling and may be useful as an analytical tool for assessing ARDS risk after cardiac surgery. It is worth noting that our dataset is specifically organized for patients who have undergone cardiac surgery. Using the SWSFS technique, we successfully identified 13 perioperative predictors. These novel predictors have the potential to allow for early detection of ARDS, personalized preventive strategies, and clinical decision-making during early stage of the disease.

The current scoring systems for critically ill patients, such as LODS, MODS, and SOFA scores, are widely used in clinical practice and have been proven to be closely associated with patient outcomes. However, these scoring systems are often subjective and incapable of effectively predicting the risk, prognosis, or mortality of patients with specific diseases. Although the ARDS, LIPS, and SLIP scores were developed for acute lung injury (ALI)/ARDS, the etiology of ARDS encompasses various factors including trauma, burns, sepsis, and pulmonary infections. These scoring systems do not incorporate disease-related pathophysiological indicators. In other words, none of these tools were ideal for ARDS in the CPB settings, making early diagnosis and personalized prevention guidance for cardiac patients difficult. Our findings indicate that the above scoring systems have insufficient discriminative ability and cannot be used to predict ARDS after cardiac surgery. Future work should focus on developing ARDS risk-scoring models within a more comprehensive and systematic cardiac database.

The predictors included in our study were a combination of clinical characteristics, laboratory biomarkers, hemodynamic parameter, and CPB process, indicating that the pathophysiology of ARDS following cardiac surgery is multifactorial and intricate. Epidemiological studies have shown that CBP is a well-known risk factor for ARDS, especially the intraoperative factors, which reflect the acute physiological response during the surgical process and play a critical role in the ARDS development. Our findings revealed that the longer the duration of CPB and ACC, the higher the risk of developing ARDS. During CPB, the pulmonary circulation stops, and the lungs receive blood supply solely from the bronchial arteries, which is insufficient to meet their metabolic demands, resulting in high oxygen consumption and increased metabolism [24]. Meanwhile, insufficient metabolic substrates reduce adenosine triphosphate production within lung tissue and increase lactate levels. The reintroduction of highly oxygenated blood to the lung tissue during aortic reperfusion disrupts the balance of superoxide dismutase and peroxidase enzymes, resulting in an abundance of oxygen free radicals. This cascade causes endothelial cell injury, increased vascular permeability, and the onset of pulmonary edema [25]. Furthermore, the systemic inflammatory response syndrome (SIRS) induced by CPB is recognized as a key factor contributing to lung injury. SIRS is initiated and progressed by activation of complement factors (C3a, C4a, and C5a), neutrophils, and an imbalance in inflammatory/anti-inflammatory cytokines (TNF-α, IL-1, IL-2, and IL-6). CPB is a predictor for the onset of ARDS and a prognostic factor for patients with ARDS [26]. CVP is a hemodynamic parameter in CPB surgery and used as an indicator of volume status and cardiac pre-load. Elevated CVP levels often reflect systemic congestion, which causes increased pulmonary circulation pressure, pulmonary edema, and vascular leakage. Intraoperative oliguria usually indicates an acute response to unstable arterial pressure and inadequate organ perfusion. These findings highlight that, during CPB, not only the importance of venous congestion but also inadequate perfusion plays a crucial role in the deterioration of lung function. The SHAP analysis identified intraoperative blood transfusion as the strongest factor influencing model output. Over the past few decades, in-depth discussions on the pathophysiology and clinical condition of transfusion-related ALI have increased [27, 28]. Perioperative blood component transfusions are generally recognized as a significant risk factor for ARDS, regardless of which component was predominantly used. In this study, we categorized blood transfusion as a multi-class variable to emphasize the influence of various blood components on ARDS. NLR has recently been reported as a potential novel biomarker of the baseline inflammatory process and could be as an outstanding predictor or prognostic marker in patients with chronic lung diseases, ARDS, and lung cancer [29–31]. NLR and WBC are relatively accessible and easy to calculate, reflecting the role of inflammation in the onset of ARDS. Taken together, our study has identified a series of novel factors contributing to the development of ARDS after cardiac surgery. These factors may reflect the pathophysiological processes of ARDS in CPB settings. Of note, some predictive factors are potentially modifiable. The use of these factors may provide a basis for early diagnosis, prevention, and treatment strategies in the preoperative and intraoperative management of patients with ARDS. These strategies might include preoperative anti-inflammatory treatment, nutrition support, intraoperative transfusion management, and optimizing hemodynamic status during CPB.

To our knowledge, published research using ML models for predicting ARDS in cardiac surgery is rare. We searched PubMed for the terms “ARDS”, “cardiac surgery”, “cardiopulmonary bypass” and “machine learning” to find English language publications reporting on the ML for predicting ARDS, covering the period from the inception of the database up to June 1st, 2023. Only two original articles were included. Using HER data, Wang et al*.* [32] applied an RF model to predict the risk of ARDS following cardiac surgery. They visualized the top 10 factors associated with the development of ARDS, some of which overlapped with the results of our work. Wang et al*.* [33] conducted a prospective nested cohort study to identify proteomic biomarkers and develop an XGBoost model for ARDS. The XGBoost calculated feature contributions for proteins at the end of CPB, yielding 11 novel protein features capable of effectively differentiating CPB-ARDS from non-ARDS cases. Other studies have reported the application of ML-based ARDS prediction in various etiologies. For example, a GBDT-based radiomics score could predict ARDS in polytraumatized patients with a sensitivity/specificity of 0.80/0.76 and an AUC of 0.79, significantly outperforming conventional trauma scores [34]. Zhang et al*.* [35] used five ML models to predict the occurrence of ARDS in patients with severe acute pancreatitis. All these models are tree-based ensemble learning algorithms. The ensemble method integrates the predictions of multiple weak classifiers to generate a strong classifier, which has the advantages of improving prediction performance, reducing errors, and enhancing the generalization ability of the models. In this study, we validated six types of ensemble learning models and proved their superiority in ARDS classification. Selecting an appropriate model is crucial for the accurate prediction of specific diseases. On the other hand, the interpretation of a ML model is also of great importance in clinical practice, as a true “black-box” can hardly be used in clinical decision-making. In recent years, a number of innovative visualization techniques such as permutation importance, Partial Dependence Plot (PDP), Local Interpretable Model-Agnostic Explanations (LIME), and SHAP, have been proposed to interpret ML models. In our study, we employed SHAP to visualize the RF model, which stood out for its ability to consider the influence of individual features and the potential synergistic effects of variable groups on the overall model. Using SHAP, we analyzed the decision paths of six individual patients, revealing the strength of each feature in distinguishing between ARDS and non-ARDS.

Before implementing ML models in clinical practice, their ability in accurate predictions on new patients from diverse clinical settings, e.g., time periods, geographical locations, or populations, should be repeatedly validated. Unlike clinical scoring systems, the ML model calculates risk without the involvement of clinicians, making it as a completely data-driven predictive tool. With advancements in EHR, the ML algorithm can be integrated into EHR systems to automatically assess the risk of ARDS by capturing important clinical features. For example, artificial intelligence models for bedside decision support can output the disease risk, identify high-risk factors, and provide text explanations: “Warning: This patient is about to go into developing ARDS.”. High-risk factors: <she had a high CVP level prior to weaning off cardiopulmonary bypass>. Recommended next steps: <conservative fluid management and closely monitoring her circulation>.

Our study has several strengths. First, we used multiple ML algorithms to predict ARDS following cardiac surgery, thus establishing the most comprehensive ML study in ARDS prediction, to the best of our knowledge. RF, XGBoost, LightGBM, and DF are particularly suitable for training with small-sample datasets. Second, we first used DF to predict ARDS after cardiac surgery. Among the 7 ML models, the DF exhibited the best model performance among seven ML models. As an alternative to deep neural networks, DF improves the robustness of traditional deep learning methods on small-scale clinical data. At the algorithmic level, DF integrates multiple RF, demonstrating a successful implementation of “second-level ensemble” in boosting the model’s predictive ability. Third, previous studies constructed ML models with all features as input variables. However, incorporating a large number of features often resulted in increased model complexity, making it challenging to validate the models on external datasets, as most features are irrelevant to ARDS classification. To address this, we employed the SWSFS technique to identify a subset of ARDS-relevant features. Including a smaller subset of features not only reduces the risk of overfitting and simplifies the model, but also facilitates model training and cross-site transportability.

We emphasize several limitations of our study. First, the training data was rather small despite the ML algorithms used in our study being more suitable for small-scale training. The ML training process is the cornerstone of model validation, interpretation, and cross-site transportability. Theoretically, ML model performance is highly dependent on data volume, and larger training dataset can improve model robustness and reduce the risk of overfitting. Second, it was a retrospective study; although the data collection process was considered high quality, the real-world clinical data were noisy, compared with synthetic datasets. The ensemble learning methods could successfully work with noisy data because this is the reality to which the models will be applied. However, a retrospective validation cohort with few positive cases (the occurrence of ARDS) limits the reliability of the validated results. External validation using prospectively collected data could address this limitation. Third, as previously stated, the issue of unbalanced data cannot be ignored. Actually, in our consecutive inpatient cohort, the incidence of ARDS was relatively low (less than 5%). The limited number of ARDS cases can potentially result in a classifier’s prediction accuracy skewed towards predicting outcomes for the larger category (patients without ARDS). To address this, we have established ARDS cohorts specifically to increase the number of positive cases and used a random oversampling technique to balance the ratio between ARDS and non-ARDS cases (nearly 1:1). However, patients who are not part of a consecutive cohort or a concurrent period may experience differences in diagnosis and treatment. In addition, the oversampling technique that overfits minority class samples may reduce their generalization ability to unseen samples.

Taken together, our study identified a set of novel predictors for CPB-induced ARDS. Meanwhile, we demonstrated the potential of tree-based ensemble methods in generating robust predictive tools for ARDS after cardiac surgery. As multicenter, multimodal EHR data systems are established, we anticipate discovering more novel associations that reflect the pathophysiology of ARDS. Future work will focus on refining clinical data structures and developing more advanced ML algorithms to aid in the risk assessment of ARDS after cardiac surgery, ultimately optimizing treatment strategies and enhancing patient prognoses.

## Conclusions

In this study, based on the EHR, we identified 13 important perioperative predictors and established multiple tree-based ensemble ML models to optimize ARDS prediction after cardiac surgery.

### Supplementary Information

Supplementary Material 1.

Supplementary Material 2.

## Data Availability

The original data supporting the findings of this study are available from the corresponding author upon reasonable request.

## Abbreviations

ARDS: Acute respiratory distress syndrome
CPB: Cardiopulmonary bypass
ML: Machine learning
EHR: Electronic health record
CABG: Coronary artery bypass grafting
ICU: Intensive care unit
OOB: Out of bag
GBDT: Gradient Boosting Decision Tree
XGBoost: EXtreme Gradient Boosting
AdaBoost: Adaptive Boosting
LightGBM: Light Gradient Boosting Machine
RF: Random Forest
DF: Deep Forest
SHAP: SHapley Additive explanation
AUC: Area under the receiver operating characteristic curve
COPD: Chronic obstructive pulmonary disease
LVEF: Left ventricular ejection fraction
LVEDD: Left ventricular end-diastolic diameter
WBC: White blood cell
NLR: Neutrophil-to-lymphocyte ratio
ACC: Aortic cross-clamping
CVP: Central venous pressure
TRALI: Transfusion-related acute lung injury
