# Supplementary material for: Tree-based ensemble machine learning models in the prediction of acute respiratory distress syndrome following cardiac surgery: a multicenter cohort study
Source: J Transl Med. 2024 Aug 15;22:772. doi: 10.1186/s12967-024-05395-1 (PMC11325832; doi:10.1186/s12967-024-05395-1)

Machine learning-based prediction of acute respiratory distress syndrome following cardiac surgery: a multicentre cohort study

Introduction to the methodology of ensemble machine learning algorithms

Ensemble learning algorithm is a method of combining multiple classifiers to make decisions. Integrating multiple weak classifiers into a strong classifier, it improves the model's performance and generalization ability. It has the advantages of increasing model accuracy and stability, improving robustness, and reducing overfitting. In this study, we employed the following tree-based ensemble learning algorithms for model construction: decision trees, GBDT, AdaBoost, XGBoost, LightGBM, Random Forest, and Deep Forests.

(i) Decision tree is a nonparametric supervised machine learning algorithm that is primarily for classification and regression problems. The decision tree divides the feature space into a limited number of disjoint subregions through a collection of if-then decision rules, giving the same prediction value for samples that fall in the same subregion. At each step of the partitioning process, the algorithm selects the feature and the threshold that best splits the data, aiming to maximize the information gain or minimize impurity in the resulting subsets. Decision trees are interpretable models that construct hierarchical decision rules, making them valuable for understanding the underlying data relationships and feature importance. However, they are prone to overfitting when the tree depth is not properly controlled, which can be mitigated using techniques like pruning or ensemble methods.

(ii) Gradient Boosting Decision Tree (GBDT) is a boosting method that utilizes an additive model where base functions are combined linearly using a forward stagewise algorithm. In GBDT, decision trees are employed as the base functions to enhance the model performance. Specifically, as an ensemble model, GBDT predicts by summing up the results of all the sub-trees. GBDT generates the entire forest by sequentially creating decision sub-trees. The process of generating a new sub-tree involves using the residuals between the sample labels and the current forest predictions to construct the new sub-tree.

(iii) Adaboost is an effective and practical Boosting algorithm. It was proposed by Freund and Schapire in 1995 as an improvement upon Boosting algorithms. The underlying principle of this algorithm involves adjusting the weights assigned to the training samples and the weights of the weak classifiers. By iteratively training weak classifiers on different sets of weighted samples, Adaboost aims to select the combination of weak classifiers with the minimum weight coefficients to construct the final strong classifier. In the Adaboost algorithm, the difficulty of classifying each sample is assessed based on the estimates provided by the previously trained weak classifiers. These difficulty assessments are then used to determine the weights assigned to the samples. During the training process, the Adaboost algorithm strategically selects the most informative features from the training set multiple times. It progressively trains a series of weak classifiers and selects the best weak classifier at each iteration using an appropriate threshold. Finally, the best weak classifiers from each iteration are combined to form the strong classifier.

(iv) XGBoost is an additive model that utilizes the boosting enhancement strategy. It works by sequentially adding weak learners (typically decision trees) to an ensemble, with each subsequent learner focusing on the mistakes made by the previous ones. The key principle behind XGBoost is gradient boosting, where the algorithm optimizes a predefined loss function by minimizing the errors between the actual and predicted values. XGBoost employs a regularization term in its objective function to control model complexity and prevent overfitting. This term penalizes large coefficients in the model, thereby promoting simpler models that generalize well to unseen data. Additionally, XGBoost utilizes a novel technique called “tree pruning” to avoid constructing an excessively deep tree, which further helps in preventing overfitting. One of the distinctive features of XGBoost is its capability to handle missing data effectively by assigning optimal values during the tree construction process. Moreover, XGBoost implements parallel computing techniques to improve training speed, making it efficient for large-scale datasets.

(v) LightGBM is a gradient-boosting framework that uses decision trees as base learners. LightGBM is designed for efficient parallel computing, and its “Light” is characterized by the following aspects: (1) faster training speed; (2) lower memory usage; (3) support for single-machine multi-threading, distributed computing, and GPU training; (4) ability to handle large-scale data. LightGBM adopts the leaf-wise growth strategy, whereby the leaf with the maximum split gain from the current set is selected for splitting, iteratively repeating this process. The advantage of this strategy is that, with the same number of splits, leaf-wise can reduce more errors and achieve better accuracy. Additionally, LightGBM also introduces a depth limit for tree growth to prevent overfitting.

(vi) Random forest is a method based on decision trees, which uses rules to binary split data. In classification tasks, the most commonly used rule to binary split data is Gini index. Decision tree methods can produce good predictions on the training set, but a tree with many splits will probably overfit and consequently lead to poor test set performance. The main problem is that the correct pruning process in many situations does not produce suitable models. In contrast, random forest uses an ensemble of decision trees without pruning and two powerful randomization processes, bagging, and random feature selection, providing more accurate results and making the model more resistant to overfitting. This machine learning algorithm became popular due to its simplicity of training and tuning parameters, the possibility to fit nonlinear models, and the production of excellent classification results.

(vii) Deep Forest is a non-neural network-based deep model characterized by a cascading structure that facilitates feature learning through the use of an ensemble of trees. It is based on non-differentiable modules that do not rely on backpropagation during the training process. Deep Forest is proposed with two ensemble components: (i) multi-gained scanning, which scans local context from high dimensionality to learn representations of input data according to different random forests; (ii) cascade forest, in which neurons of deep neural networks have been replaced with many different random forests. In Deep Forest, compared to Deep Neural Networks, fewer hyper-parameters and fewer parameter tuning skills in are required the training process. As a newly born deep learning method, the deep forest is not developed to replace deep neural networks; instead, it offers an alternative when deep neural networks are inferior to Random Forest. In this study, we used a multiple-layer cascade forest containing various RFs. This structure has been designed to ensure the diversity of the model by including different types of forests. Each layer in the cascade forest receives the information processed by the previous stage and outputs the processing results to the next layer.


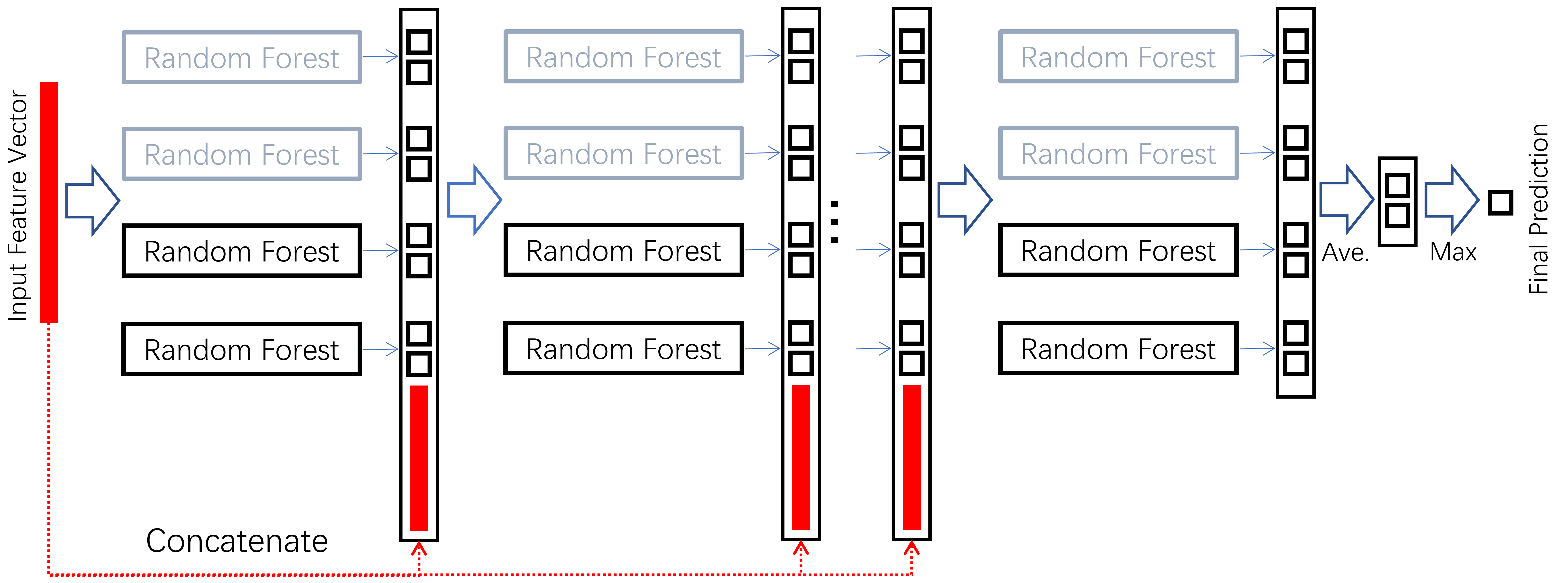

Supplement: Supplementary file 1 — Supplementary Material 1. [file 12967_2024_5395_MOESM1_ESM.docx]
