# Supplementary material for: Tree-based ensemble machine learning models in the prediction of acute respiratory distress syndrome following cardiac surgery: a multicenter cohort study
Source: J Transl Med. 2024 Aug 15;22:772. doi: 10.1186/s12967-024-05395-1 (PMC11325832; doi:10.1186/s12967-024-05395-1)
Supplement: Supplementary file 2 — Supplementary Material 2. [file 12967_2024_5395_MOESM2_ESM.docx]

Machine learning-based prediction of acute respiratory distress syndrome following cardiac surgery: a multicentre cohort study


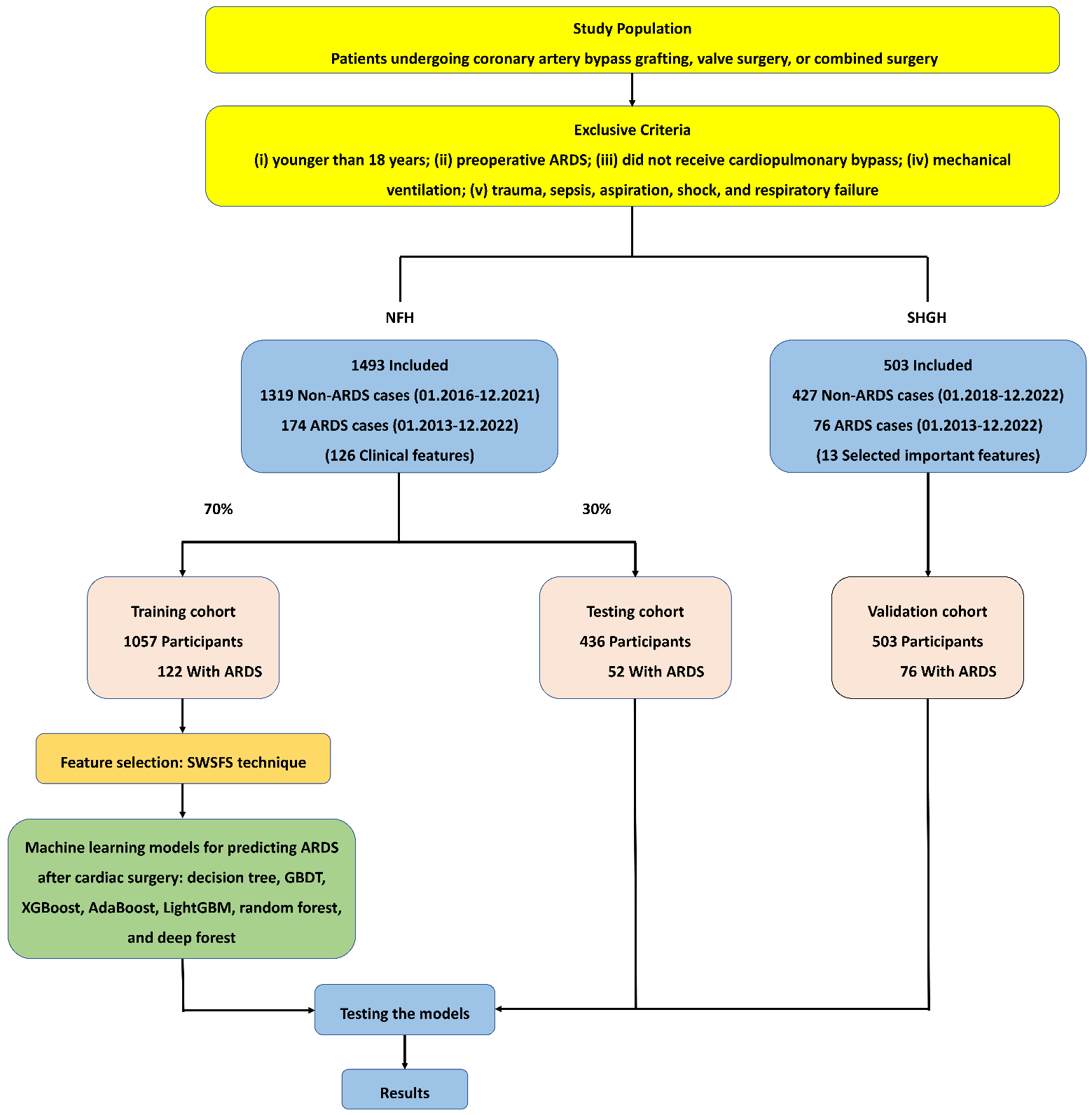


**Figure S1**. Flow chart of patient selection process and model development


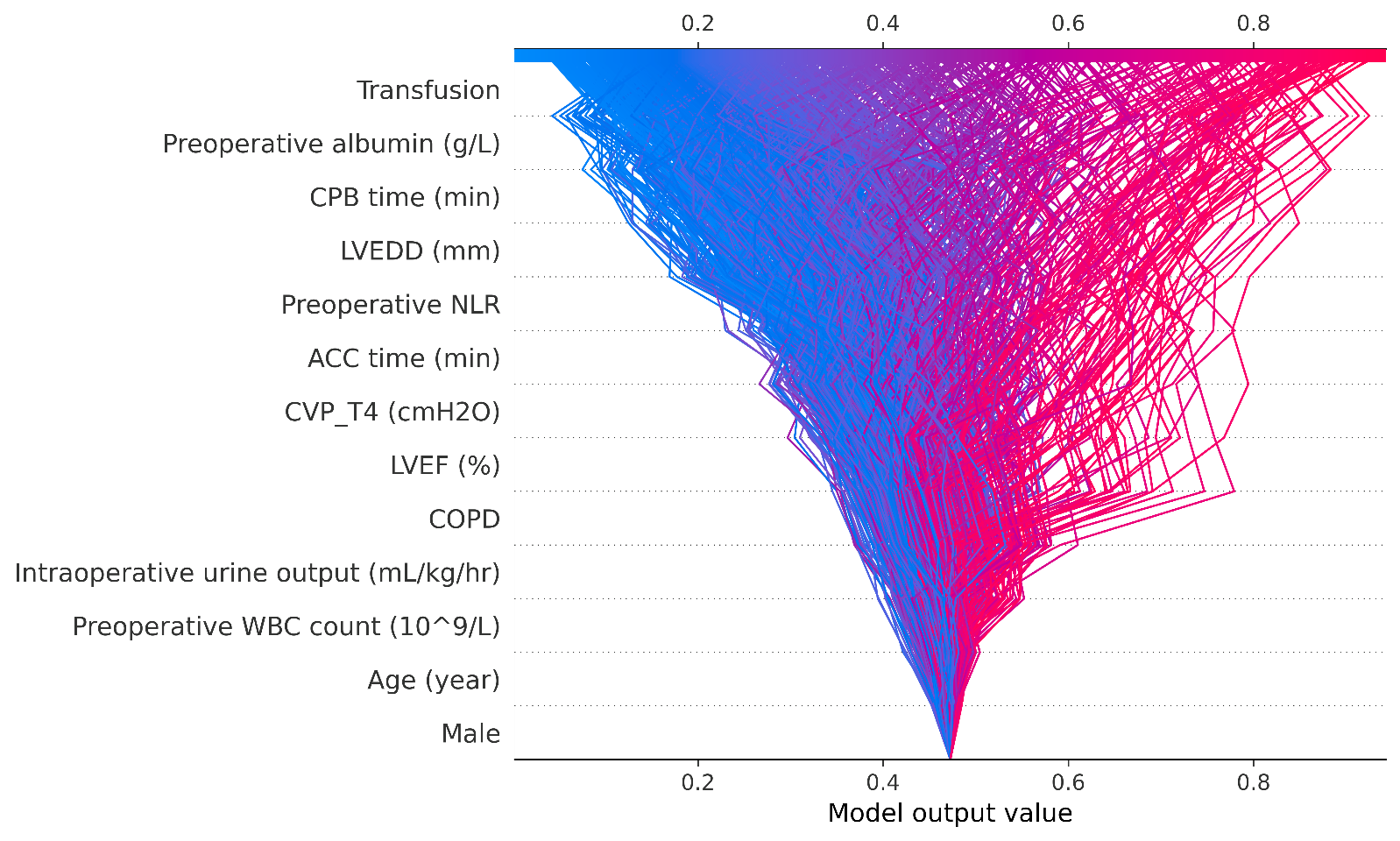


**Figure S2**. SHAP dependence plot of the random forest model. The SHAP decision plot visualizes the decision pathway for a group of patients, depicting their classification as ARDS or non-ARDS across different levels of features.


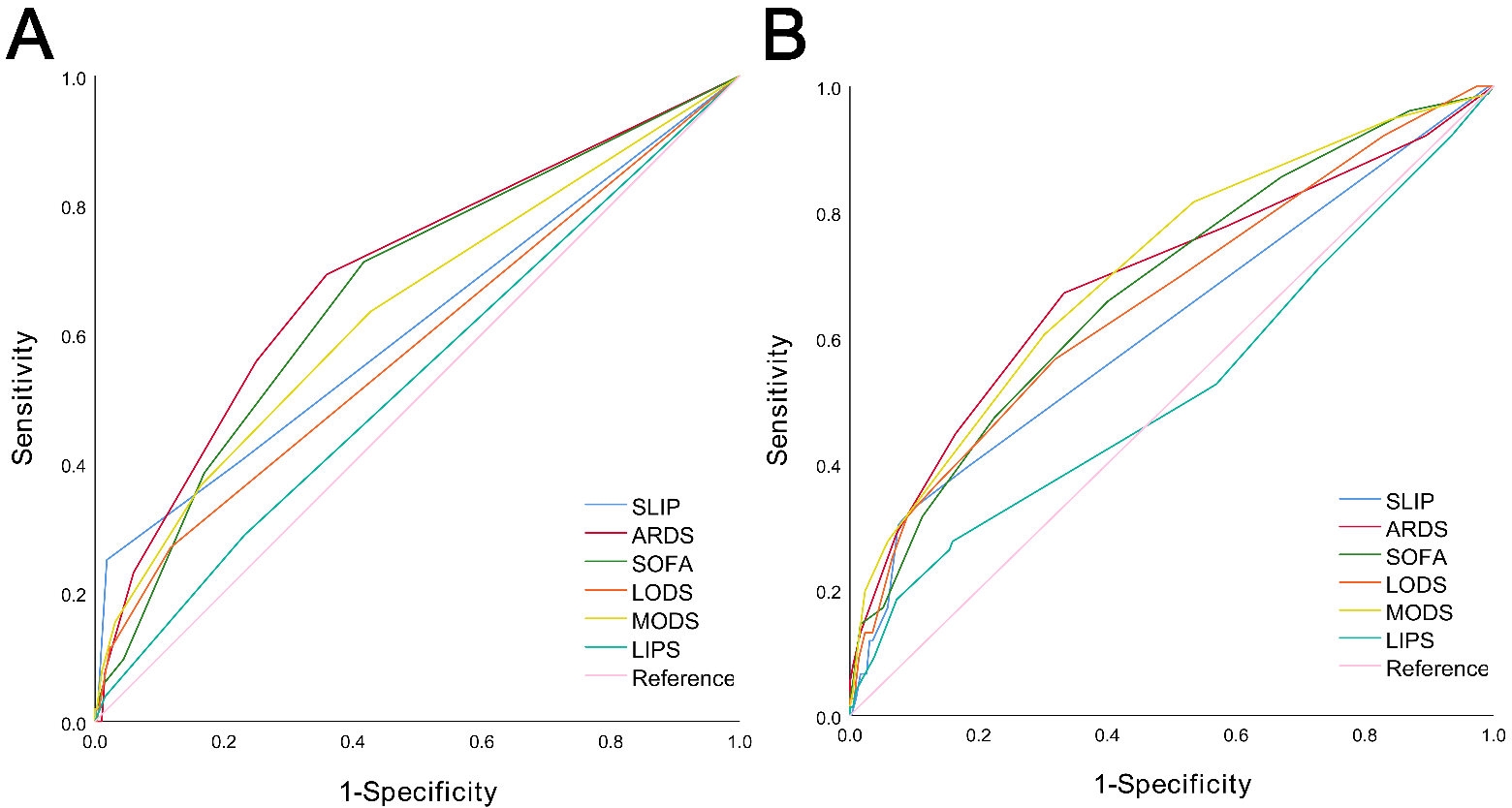


**Figure S3**. The area under the receiver operating characteristic curve of the six scoring systems for predicting ARDS in the testing (A) and validation (B) cohorts.

**Table S1**. Comparison of clinical features in training and testing cohorts.

| Candidate feature | Training Cohort  (N=1057) | | | Testing Cohort  (N=436) | | |
| --- | --- | --- | --- | --- | --- | --- |
|  | Non-ARDS  N=935 | ARDS  N=122 | p-value^*^ | Non-ARDS  N=384 | ARDS  N=52 | p-value^*^ |
| Age | 62.0 (10.0) | 64.3 (10.7) | 0.018 | 60.4 (11.1) | 65.1 (9.6) | 0.004 |
| Male | 532 (56.9%) | 86 (70.5%) | 0.006 | 215 (56.0%) | 34 (65.4%) | 0.256 |
| Rural area | 515 (55.1%) | 63 (51.6%) | 0.534 | 199 (51.8%) | 33 (63.5%) | 0.153 |
| Hepatic insufficiency | 23 (2.5%) | 2 (1.6%) | 0.762 | 8 (2.1%) | 1 (1.9%) | 1.000 |
| Hyperthyroidism | 4 (0.4%) | 1 (0.8%) | 1.000 | 5 (1.3%) | 2 (3.8%) | 0.191 |
| Hypothyroidism | 13 (1.4%) | 6 (4.9%) | 0.021 | 10 (2.6%) | 1 (1.9%) | 1.000 |
| Non statins | 23 (2.5%) | 7 (5.7%) | 0.040 | 17 (4.4%) | 1 (1.9%) | 0.508 |
| Ticagrelor | 30 (3.2%) | 6 (4.9%) | 0.425 | 8 (2.1%) | 0 (0.0%) | 0.597 |
| Insurance | 510 (54.5%) | 64 (52.5%) | 0.735 | 218 (56.8%) | 30 (57.7%) | 1.000 |
| BMI | 24.0 (3.1) | 24.3 (3.6) | 0.351 | 23.8 (3.2) | 24.5 (2.7) | 0.107 |
| Smoker | 98 (10.5%) | 20 (16.4%) | 0.072 | 33 (8.6%) | 7 (13.5%) | 0.311 |
| Diabetes mellitus |  |  | 0.521 |  |  | 0.833 |
| IDDM | 53 (5.7%) | 10 (8.2%) |  | 19 (4.9%) | 3 (5.8%) |  |
| NIDDM | 133 (14.2%) | 18 (14.8%) |  | 63 (16.4%) | 7 (13.5%) |  |
| Non | 749 (80.1%) | 94 (77.0%) |  | 302 (78.6%) | 42 (80.8%) |  |
| Hypertention | 517 (55.3%) | 62 (50.8%) | 0.402 | 197 (51.3%) | 29 (55.8%) | 0.648 |
| Hyperlipemia | 156 (16.7%) | 32 (26.2%) | 0.014 | 60 (15.6%) | 12 (23.1%) | 0.246 |
| PVD | 4 (0.4%) | 1 (0.8%) | 1.000 | 2 (0.5%) | 0 (0.0%) | 1.000 |
| COPD | 22 (2.4%) | 25 (20.5%) | <0.001 | 7 (1.8%) | 13 (25.0%) | <0.001 |
| CVA | 72 (7.7%) | 17 (13.9%) | 0.031 | 30 (7.8%) | 2 (3.8%) | 0.413 |
| IE | 16 (1.7%) | 7 (5.7%) | 0.010 | 7 (1.8%) | 2 (3.8%) | 0.601 |
| Hydrothorax | 26 (2.8%) | 5 (4.1%) | 0.579 | 7 (1.8%) | 3 (5.8%) | 0.105 |
| Angina | 399 (42.7%) | 60 (49.2%) | 0.205 | 170 (44.3%) | 25 (48.1%) | 0.712 |
| Abnormal chest X.ray | 67 (7.2%) | 27 (22.1%) | <0.001 | 18 (4.7%) | 13 (25.0%) | <0.001 |
| Previous MI | 148 (15.8%) | 29 (23.8%) | 0.037 | 57 (14.8%) | 13 (25.0%) | 0.095 |
| NYHA III-IV class | 287 (30.7%) | 62 (50.8%) | <0.001 | 93 (24.2%) | 20 (38.5%) | 0.042 |
| AF | 251 (26.8%) | 41 (33.6%) | 0.143 | 96 (25.0%) | 14 (26.9%) | 0.897 |
| Previous PCI | 61 (6.5%) | 15 (12.3%) | 0.033 | 22 (5.7%) | 3 (5.8%) | 1.000 |
| Allergy | 42 (4.5%) | 3 (2.5%) | 0.419 | 19 (4.9%) | 3 (5.8%) | 1.000 |
| Preoperative Scr | 0.8 (0.2) | 1.0 (0.4) | 0.001 | 0.9 (0.3) | 1.0 (0.4) | 0.058 |
| Preoperative TC | 4.2 (1.1) | 4.0 (1.2) | 0.076 | 4.2 (1.1) | 4.3 (1.5) | 0.846 |
| Preoperative LDL | 2.5 (0.9) | 2.5 (1.0) | 0.475 | 2.5 (0.9) | 2.7 (1.2) | 0.303 |
| CAS |  |  | 0.783 |  |  | 0.781 |
| Non | 815 (87.2%) | 108 (88.5%) |  | 351 (91.4%) | 46 (88.5%) |  |
| One side | 42 (4.5%) | 6 (4.9%) |  | 13 (3.4%) | 2 (3.8%) |  |
| Two side | 78 (8.3%) | 8 (6.6%) |  | 20 (5.2%) | 4 (7.7%) |  |
| LVDd | 436 (46.6%) | 46 (37.7%) | 0.078 | 172 (44.8%) | 27 (51.9%) | 0.412 |
| Pulmonary hypertension | 271 (29.0%) | 48 (39.3%) | 0.025 | 111 (28.9%) | 20 (38.5%) | 0.212 |
| AR | 337 (36.0%) | 63 (51.6%) | 0.001 | 133 (34.6%) | 17 (32.7%) | 0.903 |
| MR | 468 (50.1%) | 84 (68.9%) | <0.001 | 193 (50.3%) | 38 (73.1%) | 0.003 |
| TR | 350 (37.4%) | 47 (38.5%) | 0.893 | 131 (34.1%) | 27 (51.9%) | 0.019 |
| No diseased vessels |  |  | 0.794 |  |  | 0.754 |
| Non | 499 (53.4%) | 60 (49.2%) |  | 196 (51.0%) | 24 (46.2%) |  |
| One | 47 (5.0%) | 7 (5.7%) |  | 29 (7.6%) | 5 (9.6%) |  |
| Three | 348 (37.2%) | 48 (39.3%) |  | 146 (38.0%) | 20 (38.5%) |  |
| Two | 41 (4.4%) | 7 (5.7%) |  | 13 (3.4%) | 3 (5.8%) |  |
| Left main disease | 146 (15.6%) | 13 (10.7%) | 0.191 | 52 (13.5%) | 8 (15.4%) | 0.883 |
| Nitrates | 37 (4.0%) | 9 (7.4%) | 0.132 | 15 (3.9%) | 0 (0.0%) | 0.225 |
| Catecholamine | 38 (4.1%) | 10 (8.2%) | 0.067 | 15 (3.9%) | 0 (0.0%) | 0.239 |
| LMWH | 275 (29.4%) | 29 (23.8%) | 0.235 | 105 (27.3%) | 23 (44.2%) | 0.019 |
| Metoprolol | 105 (11.2%) | 24 (19.7%) | 0.011 | 46 (12.0%) | 5 (9.6%) | 0.789 |
| ACEI | 26 (2.8%) | 7 (5.7%) | 0.099 | 14 (3.6%) | 2 (3.8%) | 1.000 |
| ARB | 33 (3.5%) | 9 (7.4%) | 0.052 | 7 (1.8%) | 0 (0.0%) | 0.602 |
| Statins | 84 (9.0%) | 25 (20.5%) | <0.001 | 35 (9.1%) | 5 (9.6%) | 1.000 |
| Aspirin | 72 (7.7%) | 21 (17.2%) | 0.001 | 26 (6.8%) | 3 (5.8%) | 1.000 |
| Clopidogrel | 49 (5.2%) | 18 (14.8%) | <0.001 | 23 (6.0%) | 3 (5.8%) | 1.000 |
| Surgery type | 82 (8.8%) | 33 (27.0%) | <0.001 | 45 (11.7%) | 13 (25.0%) | 0.015 |
| Previous surgery | 10 (1.1%) | 3 (2.5%) | 0.385 | 9 (2.3%) | 0 (0.0%) | 0.401 |
| Minimally invasive approach | 37 (4.0%) | 3 (2.5%) | 0.488 | 13 (3.4%) | 1 (1.9%) | 0.718 |
| Cardioplegia type | 891 (95.3%) | 114 (93.4%) | 0.505 | 377 (98.2%) | 52 (100.0%) | 0.601 |
| Cardioplegia temperature | 890 (95.2%) | 114 (93.4%) | 0.542 | 377 (98.2%) | 51 (98.1%) | 1.000 |
| Biological valve | 250 (26.7%) | 60 (49.2%) | <0.001 | 102 (26.6%) | 19 (36.5%) | 0.179 |
| Mechanical valve | 288 (30.8%) | 29 (23.8%) | 0.136 | 126 (32.8%) | 16 (30.8%) | 0.891 |
| RFTA | 95 (10.2%) | 13 (10.7%) | 0.991 | 29 (7.6%) | 4 (7.7%) | 1.000 |
| Left auricle surgery | 66 (7.1%) | 5 (4.1%) | 0.300 | 35 (9.1%) | 4 (7.7%) | 0.801 |
| Preoperative albumin | 40.4 (3.7) | 37.7 (4.2) | <0.001 | 40.8 (3.5) | 39.0 (4.1) | 0.004 |
| Preoperative globulin | 27.7 (4.6) | 27.2 (5.0) | 0.394 | 27.8 (4.6) | 27.6 (4.9) | 0.790 |
| Preoperative AKP | 74.5 (26.6) | 78.7 (35.7) | 0.217 | 73.6 (24.5) | 80.6 (29.8) | 0.110 |
| Preoperative bilirubin | 13.6 (7.7) | 15.0 (9.7) | 0.126 | 14.1 (9.0) | 13.9 (8.0) | 0.830 |
| Preoperative TBA | 6.4 (7.0) | 5.7 (4.8) | 0.126 | 6.0 (6.8) | 6.3 (4.6) | 0.687 |
| Preoperative urea | 6.5 (2.3) | 7.5 (3.6) | 0.005 | 6.4 (2.1) | 7.2 (3.4) | 0.090 |
| Preoperative UA | 357.2 (107.5) | 399.1 (134.2) | 0.001 | 360 (109) | 402 (114) | 0.015 |
| Preoperative serum potassium | 4.0 (0.4) | 4.0 (0.4) | 0.237 | 4.0 (0.4) | 4.0 (0.5) | 0.839 |
| Preoperative serum sodium | 140.0 (2.6) | 139.5 (2.9) | 0.074 | 139.8 (2.3) | 140.2 (2.9) | 0.331 |
| Preoperative serum chloride | 101.8 (3.3) | 102.0 (3.6) | 0.535 | 101.6 (3.1) | 101.5 (3.8) | 0.843 |
| Preoperative serum calcium | 2.3 (0.1) | 2.2 (0.2) | <0.001 | 2.3 (0.1) | 2.3 (0.1) | 0.011 |
| Preoperative HDL | 1.1 (0.3) | 1.0 (0.3) | <0.001 | 1.1 (0.3) | 1.0 (0.3) | 0.114 |
| Preoperative apolipoprotein A1 | 1.3 (0.3) | 1.2 (0.3) | <0.001 | 1.3 (0.3) | 1.2 (0.3) | 0.247 |
| Preoperative apolipoprotein B | 0.8 (0.2) | 0.8 (0.3) | 0.738 | 0.8 (0.2) | 0.9 (0.3) | 0.214 |
| Preoperative lipoprotein a | 244.0 (243.2) | 259.2 (271.6) | 0.558 | 263.6 (245.8) | 328.5 (322.2) | 0.168 |
| Urine glucose | 1.1 (0.4) | 1.1 (0.4) | 0.757 | 1.1 (0.4) | 1.2 (0.6) | 0.082 |
| Urine protein | 1.1 (0.3) | 1.1 (0.3) | 0.301 | 1.1 (0.3) | 1.1 (0.3) | 0.869 |
| Urine WBC count | 28.6 (97.9) | 51.8 (179.4) | 0.162 | 40.8 (261.0) | 49.3 (169.1) | 0.754 |
| Urine RBC count | 55.7 (1314.6) | 107.0 (874.2) | 0.569 | 66.5 (583.3) | 91.0 (637.0) | 0.794 |
| Preoperative NLR | 2.5 (1.8) | 4.1 (4.8) | <0.001 | 2.5 (1.6) | 3.7 (3.2) | 0.014 |
| Preoperative LMR | 5.9 (4.8) | 4.4 (2.6) | <0.001 | 6.1 (4.7) | 7.3 (21.8) | 0.708 |
| Preoperative WBC count | 6.4 (1.9) | 7.1 (2.7) | 0.008 | 6.4 (2.0) | 7.2 (2.7) | 0.042 |
| Preoperative RBC count | 4.4 (0.6) | 4.3 (0.7) | 0.014 | 4.4 (0.5) | 4.3 (0.5) | 0.273 |
| Preoperative HCT | 40.3 (5.0) | 38.9 (5.7) | 0.014 | 40.2 (4.7) | 39.0 (4.9) | 0.085 |
| Preoperative RDW | 13.4 (1.4) | 13.9 (1.6) | <0.001 | 13.4 (1.3) | 13.9 (2.2) | 0.095 |
| Preoperative MPV | 11.3 (1.3) | 11.1 (1.2) | 0.084 | 11.2 (1.4) | 11.4 (1.1) | 0.523 |
| Preoperative PDW | 14.3 (2.9) | 13.6 (2.8) | 0.025 | 13.9 (3.0) | 14.5 (2.7) | 0.170 |
| Preoperative PT | 11.8 (3.5) | 12.0 (3.1) | 0.536 | 11.8 (3.5) | 12.4 (4.0) | 0.325 |
| Preoperative INR | 1.0 (0.3) | 1.0 (0.3) | 0.662 | 1.0 (0.3) | 1.1 (0.4) | 0.298 |
| Preoperative APTT | 28.0 (5.2) | 27.4 (6.4) | 0.347 | 28.3 (5.5) | 27.6 (7.5) | 0.530 |
| Preoperative fibrinogen | 3.0 (1.3) | 3.3 (1.6) | 0.062 | 3.0 (0.8) | 3.3 (1.2) | 0.135 |
| Preoperative D-dimer | 0.6 (1.8) | 1.1 (1.9) | 0.008 | 0.6 (1.4) | 0.7 (1.0) | 0.610 |
| Hypoglycemic agents | 109 (11.7%) | 20 (16.4%) | 0.175 | 44 (11.5%) | 9 (17.3%) | 0.324 |
| Coenzyme complex | 149 (15.9%) | 28 (23.0%) | 0.068 | 61 (15.9%) | 11 (21.2%) | 0.447 |
| Argatroban | 20 (2.1%) | 4 (3.3%) | 0.507 | 4 (1.0%) | 1 (1.9%) | 1.000 |
| Sulodexide | 12 (1.3%) | 1 (0.8%) | 0.732 | 3 (0.8%) | 0 (0.0%) | 1.000 |
| Nicotinamide | 13 (1.4%) | 4 (3.3%) | 0.116 | 6 (1.6%) | 0 (0.0%) | 0.624 |
| Intraoperative medication propofol | 660 (70.6%) | 90 (73.8%) | 0.534 | 280 (72.9%) | 42 (80.8%) | 0.298 |
| Intraoperative medication dexmedetomidine | 767 (82.0%) | 98 (80.3%) | 0.738 | 322 (83.9%) | 43 (82.7%) | 0.990 |
| Intraoperative medication ulinastatin | 176 (18.8%) | 25 (20.5%) | 0.750 | 80 (20.8%) | 10 (19.2%) | 0.932 |
| Intraoperative medication medrol | 10 (1.1%) | 2 (1.6%) | 0.638 | 4 (1.0%) | 2 (3.8%) | 0.146 |
| Intraoperative medication hydrocortisone | 84 (9.0%) | 15 (12.3%) | 0.310 | 33 (8.6%) | 7 (13.5%) | 0.301 |
| Venous cannula |  |  | 0.003 |  |  | 0.132 |
| Atria | 587 (62.8%) | 59 (48.4%) |  | 238 (62.0%) | 26 (50.0%) |  |
| Cavity | 348 (37.2%) | 63 (51.6%) |  | 146 (38.0%) | 26 (50.0%) |  |
| Arterial cannula |  |  | 0.723 |  |  | 0.636 |
| Aorta | 920 (98.4%) | 121 (99.2%) |  | 379 (98.7%) | 52 (100.0%) |  |
| Femoral | 15 (1.6%) | 1 (0.8%) |  | 5 (1.3%) | 0 (0.0%) |  |
| Potassium cardioplegia | 705 (75.4%) | 83 (68.0%) | 0.100 | 274 (71.4%) | 33 (63.5%) | 0.313 |
| Myocardial perfusion site |  |  | 0.153 |  |  | 0.441 |
| Coronary | 238 (25.5%) | 39 (32.0%) |  | 88 (22.9%) | 15 (28.8%) |  |
| Root | 697 (74.5%) | 83 (68.0%) |  | 296 (77.1%) | 37 (71.2%) |  |
| Spontaneous return | 674 (72.1%) | 89 (73.0%) | 0.926 | 280 (72.9%) | 38 (73.1%) | 1.000 |
| Intraoperative defibrillate | 1.4 (0.8) | 1.3 (0.7) | 0.674 | 1.3 (0.7) | 1.4 (0.8) | 0.708 |
| Nasopharyngeal temperature | 33.2 (0.9) | 33.0 (0.9) | 0.017 | 33.2 (0.9) | 32.9 (0.8) | 0.041 |
| Bladder temperature | 34.1 (1.0) | 33.9 (1.0) | 0.019 | 34.1 (1.0) | 33.9 (0.8) | 0.081 |
| Rectal temperature | 36.2 (0.4) | 36.2 (0.5) | 0.865 | 36.2 (0.4) | 36.3 (0.3) | 0.250 |
| Intraoperative urine output | 4.3 (4.7) | 3.0 (3.5) | 0.001 | 4.3 (4.3) | 2.8 (2.1) | <0.001 |
| Perfusion flow T3 | 1.6 (1.0) | 2.1 (0.8) | <0.001 | 1.7 (0.9) | 2.1 (0.9) | 0.002 |
| Oxygen flow T3 | 1.4 (0.9) | 1.8 (0.7) | <0.001 | 1.4 (0.8) | 1.8 (0.8) | 0.001 |
| Preoperative blood glucose | 5.5 (1.6) | 5.5 (1.4) | 0.920 | 5.4 (1.4) | 6.1 (2.2) | 0.027 |
| IABP | 7 (0.7%) | 11 (9.0%) | <0.001 | 0 (0.0%) | 1 (1.9%) | 0.111 |
| Transfusion |  |  | <0.001 |  |  | <0.001 |
| Non | 649 (69.4%) | 45 (36.9%) |  | 253 (65.9%) | 16 (30.8%) |  |
| One blood component | 193 (20.6%) | 34 (27.9%) |  | 101 (26.3%) | 22 (42.3%) |  |
| Multiple blood components | 93 (9.9%) | 43 (35.2%) |  | 30 (7.8%) | 14 (26.9%) |  |

^*^ Comparison is performed using *t* test, Mann­Whitney *U* test, chi-square test, or Fisher's exact probability method.

**Table S2**. Comparison of important features in training, testing, and validation cohorts

| Predictor | Training Cohort  N=1057 | Testing Cohort  N=436 | Validation Cohort  N=503 | P-value^*^ |
| --- | --- | --- | --- | --- |
| Male | 618 (58.5%) | 249 (57.1%) | 308 (61.2%) | 0.409 |
| Age (year) | 62.3 (10.1) | 61.0 (11.0) | 63.1 (11.6) | 0.008 |
| COPD | 47 (4.4%) | 20 (4.6%) | 42 (8.3%) | 0.004 |
| Preoperative WBC count (10^^^9/L) | 6.5 (2.0) | 6.5 (2.1) | 6.2 (2.3) | 0.021 |
| Preoperative NLR | 2.7 (2.4) | 2.6 (1.9) | 2.8 (2.4) | 0.550 |
| Preoperative albumin (g/L) | 40.1 (3.9) | 40.6 (3.6) | 37.7 (4.4) | <0.001 |
| LVEF % | 59.0 (8.6) | 59.6 (8.4) | 57.0 (8.9) | <0.001 |
| LVEDD (mm) | 53.7 (8.4) | 53.0 (8.5) | 51.8 (7.6) | <0.001 |
| CPB time (min) | 102.2 (37.8) | 100.5 (36.0) | 82.0 (54.4) | <0.001 |
| ACC time (min) | 70.4 (29.4) | 69.2 (28.7) | 52.7 (37.5) | <0.001 |
| Transfusion |  |  |  | <0.001 |
| Non | 694 (65.7%) | 269 (61.7%) | 216 (42.9%) |  |
| One blood component | 227 (21.5%) | 123 (28.2%) | 91 (18.1%) |  |
| Multiple blood components | 136 (12.9%) | 44 (10.1%) | 196 (39.0%) |  |
| CVP_T4 (cmH_2_O) | 7.3 (3.5) | 7.1 (3.4) | 6.5 (0.8) | <0.001 |
| Intraoperative urine output (mL/kg/hr) | 4.1 (4.6) | 4.2 (4.2) | 2.1 (1.3) | <0.001 |
| ARDS | 122 (11.5%) | 52 (11.9%) | 76 (15.1%) | 0.126 |

^*^ Comparison is performed using Kruskal-Wallis test or chi-square test.

**Table S3**. A detailed comparison between machine learning models and other six scoring systems

|  | ML models | SLIP | ARDS | SOFA | LODS | MODS | LIPS |
| --- | --- | --- | --- | --- | --- | --- | --- |
| End point | ARDS after cardiac surgery | ALI/ARDS after elective surgery | ARDS after cardiac surgery | sepsis-related organ failure | organ dysfunction in the ICU | multiple organ dysfunction | ALI during the hospital stay |
| Centre | multi-center | single-center | single-center | undefined | multi-center | single-center | multi-center |
| Study years | 2017-2022 | 2005-2006 | 2005-2017 | undefined | undefined | 1988-1990 | 2009 |
| Sample size | 1996 | 4366 | 2637 | undefined | 13152 | 692 | 5584 |
| External validation | ✔ | ❌ | ❌ | undefined | ✔ | ❌ | ✔ |
| AUROC in External validation | 0.643-0.827 | 0.79 | 0.78 | undefined | 0.843-0.850 | 0.928 | 0.80 |
| Features identified in the ML models | | | | | | |  |
| Male | ✔ | ❌ | ❌ | ❌ | ❌ | ❌ | ❌ |
| Age | ✔ | ❌ | ❌ | ❌ | ❌ | ❌ | ❌ |
| COPD | ✔ | ✔ | ✔ | ❌ | ❌ | ❌ | ❌ |
| Preoperative WBC count | ✔ | ❌ | ❌ | ❌ | ✔ | ❌ | ❌ |
| Preoperative NLR | ✔ | ❌ | ❌ | ❌ | ❌ | ❌ | ❌ |
| Preoperative albumin | ✔ | ❌ | ❌ | ❌ | ❌ | ❌ | ❌ |
| LVEF | ✔ | ❌ | ❌ | ❌ | ❌ | ❌ | ❌ |
| LVEDD | ✔ | ❌ | ❌ | ❌ | ❌ | ❌ | ❌ |
| CPB time | ✔ | ❌ | ✔ | ❌ | ❌ | ❌ | ❌ |
| ACC time | ✔ | ❌ | ❌ | ❌ | ❌ | ❌ | ❌ |
| Transfusion | ✔ | ❌ | ✔ | ❌ | ❌ | ❌ | ❌ |
| CVP_T4 | ✔ | ❌ | ❌ | ❌ | ❌ | ❌ | ❌ |
| Intraoperative urine output | ✔ | ❌ | ❌ | ✔ | ❌ | ❌ | ❌ |

**Table S4**. Comparison of discriminative and calibration performance of six scoring systems for predicting ARDS after cardiac surgery

| Scoring system | AUC (95% CI) | Brier score |
| --- | --- | --- |
| Testing cohort | | |
| SLIP score | 0.613 (0.521-0.705) | 0.099 |
| ARDS score | 0.692 (0.612-0.773) | 0.100 |
| SOFA score | 0.667 (0.588-0.747) | 0.101 |
| LODS score | 0.579 (0.490-0.669) | 0.102 |
| MODS score | 0.637 (0.551-0.723) | 0.100 |
| LIPS score | 0.531 (0.445-0.616) | 0.105 |
| Validation cohort | | |
| SLIP score | 0.619 (0.545-0.694) | 0.123 |
| ARDS score | 0.688 (0.616-0.760) | 0.115 |
| SOFA score | 0.678 (0.611-0.745) | 0.118 |
| LODS score | 0.661 (0.590-0.731) | 0.120 |
| MODS score | 0.708 (0.642-0.773) | 0.115 |
| LIPS score | 0.520 (0.443-0.598) | 0.128 |
